# Natural products regulate gut microbiota and SCFA metabolism in IBD: mechanisms and therapeutic potential

**DOI:** 10.3389/fmicb.2026.1942374

**Published:** 2026-10-14

**Authors:** Beihui He, Yuqian Xu, Yujin Wang, Zhiyun Pan

**Affiliations:** 1 The First Affiliated Hospital of Zhejiang Chinese Medical University (Zhejiang Provincial Hospital of Chinese Medicine), Hangzhou, China; 2 School of Life Sciences, Zhejiang Chinese Medical University, Hangzhou, China

**Keywords:** gut microbiota, inflammatory bowel disease, natural products, short-chain fatty acids, therapeutic potential

## Abstract

Inflammatory bowel disease (IBD), including ulcerative colitis (UC) and Crohn’s disease (CD), is a chronic relapsing inflammatory condition of the gastrointestinal tract. Over the past few decades, the global incidence of this disease has continued to rise, imposing a heavy socioeconomic burden. IBD is typically characterized by gut microbiota dysbiosis and reduced short-chain fatty acids (SCFAs) levels, which in turn lead to impaired intestinal barrier function and decreased immune tolerance. Multiple studies have shown that natural products, including polysaccharides, polyphenols, alkaloids, and terpenoids, can modulate the gut microbiota in IBD, with changes in SCFA concentrations observed in some preclinical studies. These effects have been associated with alterations in bacterial taxa such as *Faecalibacterium prausnitzii*, *Bifidobacterium*, and *Christensenella minuta*. These findings suggest that natural products may regulate the gut microbiota–SCFA metabolic axis; however, the direct causal relationships among changes in microbial composition, SCFA concentrations, and anti-inflammatory effects remain to be established. This review summarizes current mechanisms and translational opportunities for natural products targeting the microbiota-SCFA axis in IBD.

## 1 Introduction

Inflammatory bowel disease (IBD), which includes ulcerative colitis (UC) and Crohn’s disease (CD), comprises a group of chronic and relapsing inflammatory conditions affecting the gut. Over the past few decades, the epidemiological landscape of IBD has changed substantially (Kaplan and Windsor, 2021). Once considered a pathology of Western industrialized nations, with the highest prevalence reported in North America and Europe, IBD is now emerging as a global disease. Incidence rates are accelerating rapidly in newly industrialized regions of Asia, South America, and the Middle East, reflecting a profound influence of environmental and lifestyle factors (Ng et al., 2017). The global burden of IBD over the next 20 years will no longer be a purely medical issue, but rather a challenge requiring customized public health planning tailored to each country’s epidemiological stage (Kaplan, 2025).

The current pharmacotherapeutic armamentarium for inflammatory bowel disease encompasses aminosalicylates, corticosteroids, thiopurines, immunosuppressive agents, and biologic therapies. Although these interventions are designed to mitigate intestinal mucosal inflammation, retard disease progression, and control associated complications (Torres et al., 2017), they are not curative and are frequently compromised by primary non-response, secondary loss of response, and treatment-related adverse events (Olivera et al., 2020).

The pathophysiology of IBD is complex and multifactorial, closely related to abnormal immune responses and disruption of gut microbiota (Yang et al., 2026; Graham and Xavier, 2020). Disruption of the intestinal epithelial barrier, defective autophagy, and an imbalance between pro-inflammatory and regulatory immune pathways are central to disease pathogenesis (Garavaglia et al., 2024; Subramanian et al., 2024). However, a pivotal driver in this process is gut dysbiosis, which refers to qualitative and quantitative alterations in the composition and function of the intestinal microbiome. IBD is characterized by distinct shifts in the gut microbial community, including reduced overall microbial diversity, an imbalanced *Firmicutes*/*Bacteroidetes* ratio, and enrichment of potentially pathogenic bacterial taxa (Rausch et al., 2025). This dysbiotic ecosystem produces an altered repertoire of microbial metabolites, which are key signaling molecules and energy sources for colonic epithelial cells and immune residents (Lavelle and Sokol, 2020; Parada Venegas et al., 2019).

Among these metabolites derived from the gut microbiota, short-chain fatty acids (SCFAs), principally acetate, propionate, and butyrate, have been established as critical modulators of intestinal homeostasis (Morrison and Preston, 2016). Produced primarily by anaerobic fermentation of dietary fibers by commensal bacteria, SCFAs exert profound protective effects. Butyrate serves as the preferred energy source for colonocytes, reinforcing epithelial barrier integrity, reducing permeability, and modulating oxidative stress (Morrison and Preston, 2016; Qian et al., 2025). In parallel, SCFAs act as signaling molecules via G-protein-coupled receptors (GPR41, GPR43, GPR109a) on immune and enteroendocrine cells, promoting the differentiation and expansion of regulatory T cells (Tregs) while suppressing the activation of pro-inflammatory effectors such as NF-κB (Saadh et al., 2025). Additionally, SCFAs, particularly butyrate and propionate, inhibit histone deacetylases (HDACs), leading to anti-inflammatory effects on intestinal epithelial cells, macrophages, and dendritic cells (Dong et al., 2025). In IBD patients, fecal SCFA concentrations, especially butyrate, are consistently diminished, and this deficit correlates with active inflammation, impaired barrier function, and a reduced capacity for immune regulation (Luo et al., 2024).

The limitations of current IBD therapeutics, including primary non-response, secondary loss of response, adverse effects, and high costs, have spurred interest in microbiota-targeted strategies (Rolhion and Sokol, 2025; Xie et al., 2025). Therefore, therapeutic strategies are no longer confined to controlling inflammation, but are instead directed toward the more comprehensive goal of restoring intestinal homeostasis (Ni et al., 2017). Natural products (polyphenols, flavonoids, polysaccharides, and herbal formulations) have emerged as promising candidates in this regard. Notably, their anti-inflammatory and mucosal-healing effects appear to operate through dual mechanisms: direct immunomodulation and prebiotic-like modulation of the gut microbiome (Wang and Wu, 2021). Plant polysaccharides, for example, selectively promote SCFA-producingtaxa, particularly Bacteroidetes and Firmicutes (Franzosa et al., 2019). More specifically, certain natural compounds enrich butyrogenic genera such as *Faecalibacterium prausnitzii*, *Roseburia*, *Bifidobacterium*, *Lactobacillus*, and *Christensenella intestinihominis*, while simultaneously enhancing endogenous SCFA biosynthesis and restoring SCFA-GPR signaling (Lavelle and Sokol, 2020; Wu et al., 2026). Collectively, these findings position natural products as attractive scaffold molecules for next-generation IBD interventions, though definitive clinical validation remains forthcoming.

This article summarizes the latest research findings on how natural products affect the gut microbiota and short chain fatty acid metabolism in inflammatory bowel disease. We considered how natural compounds can fuse with SCFA mediated signaling pathways and highlighted current barriers, including low bioavailability, unclear dose-response relationships, and the gap between laboratory research results and clinical applications. Our goal is to develop microbiome targeted therapies derived from natural products, providing new ideas for the treatment of IBD.

## 2 Pathophysiological role of the gut microbiota-SCFA axis in IBD

### 2.1 Biosynthetic pathways of SCFAs and their primary producers

Short-chain fatty acid is a saturated fatty organic acid produced by microbial fermentation of undigested dietary carbohydrates, including resistant starch, dietary fiber, and oligosaccharides (Morrison and Preston, 2016). The main SCFAs in the human gut are acetic acid, propionic acid, butyric acid, valeric acid, and hexanoic acid, with acetic acid, propionic acid, and butyric acid having the highest content, accounting for over 95% of the total SCFAs. The biosynthesis of SCFA occurs through different but interrelated metabolic pathways, which vary among bacterial taxa (Ottria et al., 2026).

### 2.2 Microbial biosynthesis and metabolic cross-feeding of SCFAs

The effects of natural products on intestinal SCFA levels should be interpreted in the context of microbial metabolic pathways rather than bacterial abundance alone. Acetate, propionate, and butyrate are generated through distinct but interconnected fermentation pathways, and their production depends on substrate availability, microbial functional capacity, and metabolic interactions within the gut community (Mann et al., 2024; Deleu et al., 2021; Louis and Flint, 2017).

Acetate is widely produced by intestinal bacteria. A major route converts acetyl-CoA to acetyl-phosphate through phosphotransacetylase, encoded by pta, followed by conversion to acetate through acetate kinase, encoded by ackA (Louis and Flint, 2017; Koh et al., 2016). Acetate is not only a fermentation end product but also an important substrate for other SCFA-producing bacteria.

Propionate is mainly generated through the succinate, acrylate, and propanediol pathways. The succinate pathway is common in *Bacteroides* and related taxa and can be represented by functional genes such as mmdA. The acrylate pathway uses lactate as an intermediate and is associated with lcdA, whereas the propanediol pathway converts fucose- or rhamnose-derived 1,2-propanediol to propionate and can be identified by pduP (Culp and Goodman, 2023). These pathway-specific genes provide more direct information on propionate-producing potential than taxonomic abundance alone.

Butyrate production is more restricted and is mainly associated with bacteria such as *F. prausnitzii*, *Roseburia* spp., *Eubacterium rectale*, and *Anaerostipes* spp. (Deleu et al., 2021; Louis and Flint, 2017; Reichardt et al., 2014). In the major acetyl-CoA pathway, two acetyl-CoA molecules are converted through acetoacetyl-CoA, hydroxybutyryl-CoA, crotonyl-CoA, and butyryl-CoA. The final step occurs through two major routes. In the predominant pathway, butyryl-CoA CoA-transferase, encoded by but, converts butyryl-CoA to butyrate using acetate as a CoA acceptor. Alternatively, butyryl-CoA is converted through butyryl-phosphate and then to butyrate by butyrate kinase, encoded by buk. Metagenomic studies indicate that the but-dependent pathway is more prevalent than the buk-dependent pathway in the human gut (Reichardt et al., 2014).

A distinction should also be made between direct SCFA producers and bacteria that contribute indirectly through metabolic cross-feeding. Direct producers possess the enzymatic machinery required to synthesize and release a given SCFA, whereas cross-feeding bacteria provide intermediates such as acetate, lactate, succinate, or 1,2-propanediol that are subsequently utilized by other species (Louis and Flint, 2017; Vital et al., 2014). For example, bifidobacteria can produce acetate and lactate, which may then support butyrate production by *F. prausnitzii* and *Anaerostipes* spp. (Rios-Covian et al., 2015). Thus, SCFA production is better viewed as a community-level metabolic function rather than a property of individual taxa.

This distinction is particularly relevant when evaluating natural products in IBD. Polysaccharides may act as fermentable substrates, whereas polyphenols, flavonoids, alkaloids, and saponins may reshape microbial communities and cross-feeding networks (Cheng et al., 2024; Yao et al., 2024). Therefore, increased fecal SCFA levels after natural-product treatment may reflect expansion of direct producers, enhanced expression of functional genes such as but or buk, increased availability of metabolic intermediates, or altered host absorption. Importantly, 16S-based increases in putative SCFA-producing taxa do not necessarily demonstrate increased SCFA biosynthesis. In IBD, inflammation may also impair epithelial uptake and utilization of butyrate, meaning that fecal SCFA concentrations do not directly represent microbial production (Lloyd-Price et al., 2019; Ferrer-Picón et al., 2020). Future studies should therefore combine pathway-specific genes, metagenomics or metatranscriptomics, targeted SCFA metabolomics, and, where possible, isotope tracing to establish whether natural products truly enhance SCFA biosynthetic flux.

### 2.3 Receptor-dependent and receptor-independent mechanisms of SCFAs

Short-chain fatty acids regulate intestinal immunity through both G protein-coupled receptor (GPCR)-dependent and receptor-independent mechanisms. GPR41 (FFAR3) and GPR43 (FFAR2) are major SCFA-responsive receptors expressed in intestinal epithelial and immune cells. However, their biological effects should not be regarded as uniformly anti-inflammatory. The outcome of GPR41/GPR43 signaling depends on the receptor subtype, responding cell type, ligand concentration, tissue location, inflammatory context, and experimental model (Mann et al., 2024; Lee et al., 2024; Sun et al., 2017).

Several studies support a homeostatic or protective role of SCFA receptor signaling in the intestine. GPR41/GPR43 activation can modulate epithelial and immune-cell responses and contribute to mucosal immune regulation. However, apparently contradictory findings have also been reported. Kim et al. (2013) showed that SCFA-induced activation of GPR41 and GPR43 in intestinal epithelial cells promoted ERK1/2- and p38-dependentchemokine and cytokine production. GPR41- or GPR43-deficient mice exhibited reduced inflammatory responses in ethanol- and TNBS-induced injury models, but also showed delayed immune responses and impaired clearance of Citrobacter rodentium (Kim et al., 2013). Thus, GPR41/GPR43 signaling may facilitate protective immune recruitment during infection while also amplifying tissue inflammation under certain conditions. These findings indicate that the function of these receptors is context dependent rather than intrinsically anti-inflammatory.

Short-chain fatty acids also exert substantial receptor-independent effects. Butyrate and propionate can enter cells and inhibit histone deacetylases (HDACs), thereby altering histone acetylation and transcriptional programs involved in immune-cell differentiation and inflammatory regulation. HDAC inhibition contributes to the regulation of FoxP3^+^ regulatory T cells and other immune-cell functions (Calvo-Barreiro et al., 2023).

Similarly, modulation of NF-κB and NLRP3 should not automatically be interpreted as evidence of GPR41/GPR43 activation. These inflammatory pathways may represent downstream convergence points affected through receptor-dependent signaling, HDAC inhibition, changes in cellular metabolism, or combinations of these mechanisms. SCFAs also exert direct metabolic effects after cellular uptake. Butyrate is a major oxidative substrate for colonocytes and influences cellular energy metabolism, mitochondrial function, and autophagy (Donohoe et al., 2011).

This distinction is particularly important when evaluating natural products in IBD. Many preclinical studies report simultaneous increases in SCFAs, GPR41/GPR43 expression, and suppression of NF-κB or NLRP3. However, current evidence is still insufficient to establish whether the effects of natural products are truly dependent on specific SCFA receptors, as most studies rely on changes in receptor expression rather than direct functional validation. Future studies using receptor knockout models, selective pharmacological blockade, and functional rescue approaches are expected to clarify receptor dependency and provide a more rigorous framework for evaluating SCFA-mediated mechanisms in IBD.

### 2.4 Immunoregulatory functions of SCFAs

The immune regulatory function of SCFAs is mediated through receptor-dependent signaling and epigenetic modifications. As ligands of GPR41 and GPR43, these two receptors are expressed in epithelial cells, endocrine cells, and various immune cells. SCFAs activate downstream cascade reactions, inhibit the production of pro-inflammatory cytokines, and indirectly regulate immune homeostasis through GPR41 (Lee et al., 2024). Reduced levels of SCFA in patients with inflammatory bowel disease (IBD) can impair GPR43 signaling, leading to persistent inflammation (Tan et al., 2016). Butyric acid and propionic acid inhibit NF-κB by blocking its degradation, thereby limiting the transcription of TNF-α, IL-6, and IL-1β (Huang et al., 2020). SCFA promotes Treg differentiation of FoxP3^+^ through HDAC inhibition and GPR43 signaling, while inhibiting Th17 cells by suppressing IL-6/IL-23 production and RORγt expression (Calvo-Barreiro et al., 2023; McBride et al., 2023). In IBD, this Treg/Th17 balance tends toward Th17 dominance; supplementing SCFA provides a new therapeutic strategy for restoring mucosal tolerance and controlling chronic inflammation (Calvo-Barreiro et al., 2023; McBride et al., 2023).

### 2.5 Dysbiosis and SCFA deficiency in IBD

The intestinal microbiome of patients with IBD is characterized by a profound and reproducible alteration in community composition and metabolic output, collectively termed dysbiosis. Among the most consistent and functionally significant hallmarks of this dysbiosis is the selective depletion of SCFA-producingbacteria and a corresponding reduction in fecal and luminal SCFA concentrations—a deficit that directly links microbial ecology to disease pathogenesis. Among the most consistent features of this dysbiosis is the depletion of taxa with putative SCFA-producingcapacity and a corresponding reduction in fecal and luminal SCFA concentrations, which are frequently associated with intestinal inflammation and disease activity.

Reduction in SCFA-producingbacteria. A substantial body of evidence from metagenomic and 16S rRNA sequencing studies has demonstrated that the gut microbiota of IBD patients harbors significantly lower abundances of butyrate-producing taxa within the phyla Firmicutes and Bacteroidetes. Specifically, key butyrate producers such as *F. prausnitzii*, *Roseburia* spp. (including *R. hominis* and *R. inulinivorans*), *Eubacterium rectale*, and *Eubacterium hallii* are consistently depleted in both fecal and mucosal samples from patients with active Crohn’s disease and ulcerative colitis (Frank et al., 2007; Sokol et al., 2008; Geirnaert et al., 2017). Similarly, propionate-producing taxa, including *Bacteroides* spp., *Phascolarctobacterium* spp., and *Dialister* spp., are also reduced, while acetate producers such as *Bifidobacterium* and *Lactobacillus* show variable but often diminished abundance (Ni et al., 2017; Franzosa et al., 2019).

Reduction in fecal and luminal SCFAs (acetate, propionate, butyrate). Consistent with the compositional depletion of SCFA-producingbacteria, metabolomic profiling of fecal samples from IBD patients has revealed significantly lower concentrations of acetate, propionate, and butyrate compared to healthy controls (Machiels et al., 2014; Huda-Faujan et al., 2010). This SCFA deficit is particularly pronounced during active disease flares and correlates with key clinical and pathological parameters, including higher fecal calprotectin levels, endoscopic disease activity scores, and increased intestinal permeability (Gerasimidis et al., 2020; Marchesi et al., 2016).

Experimental studies indicate that an increase in SCFA availability does not necessarily result in an improvement in colitis severity. In a murine dextran sulfate sodium (DSS)-induced colitis model, supplementation with sodium butyrate or a mixture of SCFAs altered gut microbial composition and T-cell responses, including increases in CD4+Foxp3+ regulatory T cells and IL-17-producing T cells; however, these interventions did not significantly improve body weight change, colonic length, or histological inflammation scores compared with DSS-treated controls (Lee et al., 2022). These findings indicate that changes in SCFA availability and immune responses do not necessarily translate into measurable therapeutic effects, highlighting the limitation of inferring treatment efficacy from SCFA alterations alone. Conversely, intervention-based studies provide stronger evidence when the microbiota or specific microbial taxa are experimentally manipulated. For example, in a DSS-induced ulcerative colitis model, paeonol treatment was associated with an increase in *Clostridium butyricum* and SCFA production together with improved intestinal barrier integrity. Importantly, antibiotic-mediated depletion of the gut microbiota abolished the paeonol-associated increase in SCFA production and barrier repair, whereas subsequent *C. butyricum* colonization restored these effects (Zhao et al., 2023). Such findings provide stronger experimental support for a microbiota-dependent contribution to SCFA-related therapeutic effects than studies based solely on correlations between bacterial abundance, SCFA concentrations, and disease activity. Nevertheless, evidence from specific animal models should not be generalized to all SCFA-producingtaxa, SCFA metabolites, or natural products.

Functional consequences of SCFA deficiency in IBD. The depletion of SCFAs has profound functional implications that extend beyond energy deficiency for colonocytes. Reduced butyrate availability compromises epithelial barrier integrity, as colonocytes deprived of their primary metabolic fuel resort to anaerobic glycolysis and increased oxidative stress, leading to disrupted tight junctions and increased permeability (Donohoe et al., 2011; Wang et al., 2012). Concurrently, the diminished SCFA pool fails to adequately activate GPR41/GPR43-mediated anti-inflammatory signaling and HDAC inhibition, resulting in unchecked NF-κB activation, NLRP3 inflammasome assembly, and the persistence of a pro-inflammatory cytokine milieu (Segain et al., 2000; Yuan et al., 2020). Moreover, the Treg/Th17 balance remains skewed toward inflammation in the absence of sufficient SCFA-driven Treg differentiation, and the mucus layer becomes thinner and more penetrable due to insufficient MUC2 synthesis (Wrzosek et al., 2013; Smith et al., 2013).

Taken together, depletion of taxa with SCFA-producingcapacity and reduced fecal or luminal acetate, propionate, and butyrate are consistently observed features of intestinal dysbiosis in IBD and are frequently associated with disease activity. Experimental studies further support biological roles for SCFAs in regulating epithelial barrier integrity and immune responses. However, the extent to which reduced SCFA availability represents a primary driver of IBD pathogenesis, rather than a consequence of intestinal inflammation, altered dietary intake, accelerated intestinal transit, or other disease-related factors, remains incompletely defined. Moreover, changes in SCFA concentrations do not necessarily predict therapeutic efficacy, as demonstrated by experimental SCFA supplementation studies. Therefore, the causal contribution of the microbiota–SCFA axis should be evaluated using integrated evidence that combines microbial composition, functional pathway analysis, direct SCFA measurements, metabolic activity or flux, and targeted microbial, metabolic, or receptor-level interventions.

## 3 Natural products targeting the gut microbiota–SCFA axis

Given the central role of SCFA deficiency in IBD pathogenesis, restoring intestinal SCFAs levels through exogenous intervention has emerged as a promising therapeutic strategy. Natural products, including polyphenols, flavonoids, alkaloids, polysaccharides, and complex herbal formulations, have gained considerable attention as modulators of the microbiota-SCFA axis due to their structural diversity and broad biological activities (Table 1). Unlike direct immunosuppressants, many natural compounds regulate gut microbial composition and metabolic function through prebiotic-like mechanisms, thereby indirectly restoring intestinal homeostasis (Figure 1).

**TABLE 1:** Representative natural-product categories and their microbiota- and short-chain fatty acid (SCFA)-modulating properties.

Natural-product category	Representative compounds/sources	Microbiota modulation	SCFA-related effects
Polyphenols	Curcumin, resveratrol, gallic acid; flavonoids such as catechins/EGCG, quercetin, apigenin, luteolin, and soy isoflavones	Suppress potential pathobionts and promote beneficial or SCFA-associated bacteria, including *Lactobacillus*, *Bifidobacterium*, *Bacteroides*, *Roseburia*, and *Faecalibacterium*	Increase total SCFA availability, particularly butyrate, with compound-dependent effects on acetate and propionate
Polysaccharides	Lycium barbarum polysaccharide, *Astragalus* polysaccharide, ginseng polysaccharides	Act as fermentable/prebiotic-like substrates and promote SCFA-associated bacteria such as *Bifidobacterium*, *Lactobacillus*, *Roseburia*, and *Faecalibacterium*	Increase acetate, propionate, and butyrate; may also enhance substrate supply and microbial cross-feeding
Alkaloids	Berberine, matrine	Suppress expansion of potential pathobionts and modulate the relative abundance of SCFA-associated taxa	Restore SCFA profiles, particularly butyrate and propionate, although pathway-specific evidence remains limited
Terpenoids	Ginsenosides (e.g., Rb3 and Rk2), astragaloside IV, ursolic acid	Remodel dysbiotic microbial communities and promote beneficial or SCFA-associated taxa; effects vary among individual terpenoids and experimental models	Several preclinical studies report restoration of SCFAs, including acetate, propionate, and butyrate; direct effects on SCFA biosynthetic pathways require further validation
Complex herbal formulations	Gegen Qinlian decoction, Huanglian Jiedu decoction, Wumei Wan	Multicomponent modulation of microbial community structure and ecological interactions	May enhance overall SCFA production or restore altered SCFA profiles through multiple microbial targets

**FIGURE 1:**
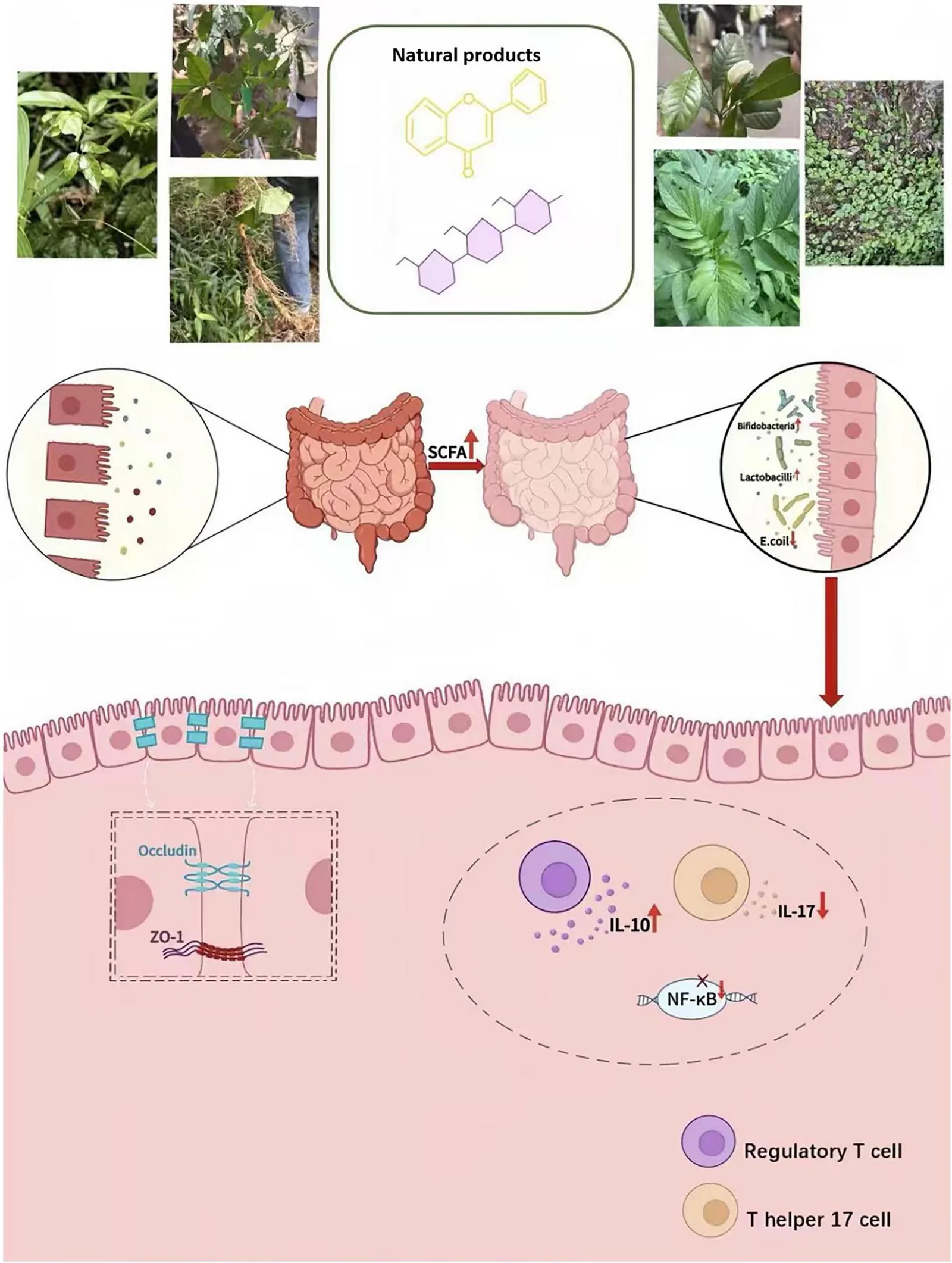
Mechanisms by which natural products ameliorate inflammatory bowel disease (IBD) through modulation of the gut microbiota- short-chain fatty acid (SCFA) axis.

Natural products (polyphenols, flavonoids, polysaccharides, and herbal formulations) exert anti-inflammatory effects by restoring gut microbial ecology and SCFA metabolism. Microbiota remodeling: Selectively enrich SCFA-producingbacteria (*F. prausnitzii*, *Roseburia*, *Bifidobacterium*) while suppressing facultative anaerobes. SCFA signaling restoration: SCFAs activate GPR41/GPR43 to inhibit pro-inflammatory cytokines; concurrently inhibit HDAC, downregulate NF-κB pathway, and promote Treg differentiation. Barrier repair and immune homeostasis: Upregulate tight junction proteins and MUC2 to reinforce epithelial barrier; restore Treg/Th17 balance and suppress NLRP3 inflammasome activation.

### 3.1 Regulation of SCFA metabolism by polyphenols

Approximately 90% of dietary polyphenols reach the colon intact, where they engage in bidirectional interactions with the gut microbiota (Cheng et al., 2023; Wang et al., 2024). Curcumin, resveratrol, and EGCG have been demonstrated to promote the proliferation of SCFA-producingbacteria, including *F. prausnitzii*, *Roseburia* spp., and *Eubacterium rectale*, while inhibiting opportunistic pathogens (Cheng et al., 2023; Ismail et al., 2025). These compositional shifts have been associated with increased fecal butyrate and propionate concentrations in experimental models. However, changes in the abundance of putative SCFA-producingtaxa and fecal SCFA concentrations do not by themselves establish increased microbial SCFA production or demonstrate that the observed anti-inflammatory effects are mediated specifically through GPR43 (Meiners et al., 2025; Wang et al., 2024).

### 3.2 Regulation of SCFA metabolism by polysaccharides

Plant polysaccharides, widely present in traditional Chinese medicine, exhibit prebiotic-like properties that promote the selective proliferation of SCFA-producingbacteria. Lycium barbarum polysaccharide (LBP) has been reported to increase the abundance of taxa associated with butyrate production, including *Roseburia* and *Faecalibacterium*, and to increase fecal butyrate and propionate concentrations in DSS-induced colitis models. These changes were accompanied by reduced NF-κB and NLRP3 inflammasome activation; however, the available evidence does not necessarily establish that these effects are mediated exclusively through the SCFA–GPR43 pathway (Wang et al., 2020). Astragalus polysaccharide (APS) promotes *Bifidobacterium* and *Lactobacillus* proliferation, upregulates but and buk gene expression, and enhances FoxP3^+^ Treg differentiation through HDAC inhibition (Zhang et al., 2019; Li et al., 2020).

### 3.3 Regulation of SCFA metabolism by alkaloids

Berberine, the best-studied alkaloid, exerts a dual modulatory effect: it suppresses pathobionts (*Escherichia coli*, *Enterococcus faecalis*) while promoting SCFA-producinggenera (*Bacteroides*, *Roseburia*, *Faecalibacterium*). Berberine intervention restores fecal butyrate and propionate levels, upregulates GPR43 expression, and suppresses NLRP3 inflammasome-mediated IL-1β/IL-18 maturation in experimental colitis (Zhang et al., 2015; Dong et al., 2020).

### 3.4 Regulation of SCFA metabolism by terpenoids

Terpenoids have also been reported to modulate the microbiota–SCFA axis in experimental colitis. Ginsenoside Rb3 alleviated DSS-induced colitis and was accompanied by increased *Lactobacillus*, decreased *Bacillus*, and higher acetate and butyrate levels, together with improved tight-junction protein expression and reduced inflammatory cytokines (Zhang et al., 2026). More mechanistic evidence has been reported for astragaloside IV (AS-IV), which remodeled gut microbiota, enhanced butyrate metabolism, and restored the Th17/Treg balance in chronic DSS colitis. Notably, microbiota depletion markedly weakened these effects, whereas fecal microbiota transplantation from AS-IV-treated mice restored colitis protection, butyrate metabolism, and Th17/Treg regulation (Zhong et al., 2026). Ursolic acid (UA), a pentacyclic triterpenoid, has also shown microbiota-related activity in DSS-induced colitis. UA treatment alleviated intestinal inflammation and restored the Treg/Th17 balance, which was accompanied by alterations in gut microbiota and potentially increased propionate production by SCFA-producing bacteria (Zhao et al., 2025). These findings suggest that some terpenoids may act through microbiota-dependent SCFA metabolic networks. However, direct regulation of specific SCFA biosynthetic pathways or metabolic flux remains insufficiently demonstrated.

### 3.5 Regulation of SCFA metabolism by natural herbal products

Traditional herbal formulas such as Gegen Qinlian decoction and Wumei Wan have shown superior efficacy compared to single compounds in restructuring gut microbiota and restoring SCFA metabolism, likely through multi-component synergistic effects (Wang and Wu, 2021; Duan et al., 2026).

### 3.6 Mechanisms by which natural products regulate gut microbiota-SCFA axis in IBD

Studies of polyphenols, polysaccharides, alkaloids, terpenoids, and herbal formulas suggest that natural products can modulate the gut microbiota–SCFA axis in experimental IBD. Reported changes include enrichment of taxa associated with SCFA production, alterations in SCFA-related genes, and changes in fecal or luminal acetate, propionate, and butyrate levels. However, these findings do not necessarily indicate increased SCFA production, as microbial abundance and functional gene changes do not directly reflect metabolic activity. Likewise, fecal SCFA concentrations are affected by intestinal absorption and host metabolism and therefore cannot fully represent in situ SCFA production. Thus, current evidence supports a potential role of the gut microbiota–SCFA axis in the effects of natural products, but its causal contribution to the improvement of IBD remains to be established (Table 2).

**TABLE 2:** Mechanistic levels of natural-product regulation of the gut microbiota–short-chain fatty acid (SCFA) axis in inflammatory bowel disease (IBD).

Mechanistic level	Specific mechanism	Representative natural products	Evidence and interpretation
Microbial composition	Enrichment of putative SCFA-producing bacteria	Polysaccharides, polyphenols	Taxonomic enrichment alone does not demonstrate increased SCFA biosynthetic activity
SCFA biosynthesis	Regulation of microbial SCFA-producing pathways, including *but/buk*-associated butyrate synthesis	*Astragalus* polysaccharide, berberine	Functional genes or pathway-level measurements provide stronger evidence than 16S abundance
Metabolic cross-feeding	Provision of acetate, lactate, or other intermediates to downstream SCFA producers	Fermentable polysaccharides	SCFA production may arise from community-level metabolic cooperation rather than expansion of a single taxon
GPR41/GPR43-dependent signaling	SCFA sensing through FFAR3/FFAR2 in epithelial and immune cells	LBP, curcumin	Effects are receptor-, cell-, tissue-, and model-dependent; receptor expression does not establish receptor activation
Receptor-independent epigenetic regulation	HDAC inhibition and altered transcriptional regulation	Butyrate; natural products that increase butyrate availability	Mechanistically distinct from GPR41/GPR43 signaling
Inflammatory pathway regulation	Modulation of NF-κB and NLRP3	Berberine, LBP	These pathways may be regulated through both GPR-dependent and GPR-independent mechanisms; receptor dependency should not be assumed

Although several clinical studies have evaluated natural products in patients with IBD, most have not simultaneously assessed gut microbiota and SCFAs. Recent clinical evidence integrating microbiota and metabolite analyses has reported changes in microbial composition or SCFA-related metabolites following natural-product interventions; however, these findings remain largely associative and do not establish that microbiota–SCFA modulation directly mediates clinical improvement (Table 3). Further clinical studies integrating microbial functional pathways, SCFA measurements, and clinical outcomes are needed to clarify the translational relevance of the microbiota–SCFA axis in IBD.

**TABLE 3:** Human clinical evidence for natural products in inflammatory bowel disease (IBD) and their effects on the gut microbiota–short-chain fatty acid (SCFA) axis.

Natural product/ intervention	IBD	Study design	Dose/ duration	Clinical outcomes	Microbiota/SCFA findings	Reference
Curcumin	UC	RCT, *n* = 70	1500 mg/day, 8 week	Clinical and inflammatory improvements	Not assessed	Sadeghi et al., 2020
Resveratrol	UC	RCT, *n* = 50	500 mg/day, 6 week	Reduced inflammatory activity	Not assessed	Samsami-Kor et al., 2015
Berberine	UC	Phase I RCT, *n* = 16	900 mg/day, 3 months	Safety and tissue inflammatory outcomes	Not assessed	Xu et al., 2020
Curcumin-QingDai	UC	RCT, *n* = 42	3 g/day, 8 week	Clinical and endoscopic improvements	Not assessed	Ben-Horin et al., 2024
Chinese herbal therapies	UC	Multicenter RCTs, *n* = 143	Disease-specific regimens	Clinical improvements reported	Microbiota and SCFA changes	Li N. et al., 2025
Xanthohumol	CD	Phase II RCT, *n* = 19	24 mg/day, 8 week	CDAI-related changes	Microbiota metabolism; acetate increased	Jamieson et al., 2026

## 4 Critical mechanistic and translational considerations

Despite compelling preclinical evidence establishing natural products as promising modulators of the gut microbiota–SCFA axis in IBD, several critical challenges must be addressed to translate these findings into clinical practice. These challenges span fundamental mechanistic understanding, pharmaceutical development, and computational biology, requiring multidisciplinary collaboration.

### 4.1 The need for mechanistic depth

Although many studies have linked natural product treatment with changes in SCFAs levels and improvement of experimental colitis, the causal relationship between these findings remains unclear. In particular, changes in the abundance of SCFA-associated bacteria or in SCFA-related genes do not necessarily indicate increased SCFA production, and fecal SCFA concentrations may not accurately reflect SCFA production at the intestinal site. These limitations make it difficult to determine whether changes in SCFA metabolism are directly responsible for the anti-inflammatory effects of natural products or occur alongside other biological effects.

Validation through GPR41/GPR43-specific agonists or knockout models. SCFA-mediated effects involve signaling through GPR41 (FFAR3) and GPR43 (FFAR2), but also include receptor-independent mechanisms such as histone deacetylase (HDAC) inhibition. The relative contribution of these pathways to the effects of natural products remains uncertain. Future studies should employ GPR41- and GPR43-deficient mouse models (constitutive or conditional knockouts) to better determine receptor dependency (Brown et al., 2003; Kim et al., 2013). Complementary use of selective small-molecule agonists or antagonists targeting these receptors would enable precise dissection of GPR41- versus GPR43-specific pathways and their distinct contribution Ws to mucosal homeostasis (Sun et al., 2017). Such approaches will also clarify whether natural products exert immunomodulatory effects primarily through direct SCFA receptor engagement or via alternative routes such as HDAC inhibition or metabolic reprogramming of immune cells.

Regional differences in metabolism and responses along the intestinal tract. A major gap in current knowledge is the regional heterogeneity of SCFA metabolism along the proximal–distal axis. The colon and ileum differ markedly in microbial composition, oxygen tension, SCFA concentrations, and receptor expression profiles. Butyrate concentrations are significantly higher in the cecum and proximal colon compared with the ileum, while GPR43 expression is more prominent in the ileum and GPR41 in the colon (Brown et al., 2003; Wang et al., 2017). Moreover, the anatomical distribution of inflammation differs between Crohn’s disease (frequently affecting the terminal ileum) and ulcerative colitis (restricted to the colon). Therefore, therapeutic responses to natural products may differ between diseases and among inflammatory phenotypes. Future investigations should systematically map SCFA production and receptor activation along the intestinal axis following natural product intervention, utilizing regional sampling (ileal versus colonic) in animal models and, where feasible, human biopsy-derived organoids.

### 4.2 Clinical translation barriers

The translation of natural products from bench to bedside is hindered by pharmaceutical and clinical obstacles requiring innovative solutions.

Low bioavailability and the need for targeted delivery. Dose-response relationships and inter-individual variability represent additional barriers to translation. The biological effects of natural products may depend on dose, treatment duration, and the baseline metabolic capacity of the gut microbiota. Importantly, a higher dose does not necessarily produce a greater beneficial effect, particularly when pharmacokinetic exposure, microbial metabolism, or host tolerance becomes limiting. Inter-individual differences in gut microbial composition can also alter the biotransformation and biological activity of natural products, potentially contributing to heterogeneous responses among patients (Espín et al., 2017). Therefore, dose optimization should be considered together with baseline microbiome characteristics and microbial metabolic capacity rather than relying solely on administered dose. The majority of polyphenols, flavonoids, and alkaloids share common pharmacokinetic limitations, including poor aqueous solubility, Qlimited intestinal permeability, extensive first-pass metabolism, and rapid hepatic clearance. Consequently, their plasma concentrations following oral administration are typically orders of magnitude lower than the effective concentrations observed *in vitro* (Dai et al., 2019). Curcumin provides a representative example: despite potent bioactivity in cell-based and animal models, its systemic exposure after oral administration is low, while its local intestinal exposure and microbial biotransformation may contribute to its biological effects (Anand et al., 2007).

To overcome these barriers, a variety of formulation approaches have been developed, such as nanoparticle encapsulation, liposomal carriers, solid lipid nanoparticles, phytosome complexes (e.g., Meriva™), and co-administration with absorption enhancers like piperine (Shen et al., 2020). Nevertheless, head-to-head comparative studies that systematically evaluate the relative efficacy of these strategies—particularly with respect to targeted delivery of active compounds to the colon, where gut microbiota modulation primarily occurs—remain conspicuously absent. Future investigations should therefore prioritize the design and optimization of colon-specific delivery systems capable of releasing natural products in the lower gastrointestinal tract.

Beyond bioavailability, long-term safety data are equally insufficient. Although natural products are often perceived as inherently safe, chronic administration, especially at concentrated or supra-physiological doses, may entail appreciable risks, including herb–drug interactions, hepatotoxicity, nephrotoxicity, and unintended modulation of the gut microbiota that could potentially favor dysbiosis (Posadzki et al., 2013; Liu et al., 2020). Moreover, safety cannot be inferred solely from the absence of acute toxicity, as long-term effects and interactions with concomitant IBD therapies remain insufficiently characterized. Given that IBD patients frequently receive concomitant immunosuppressive or biologic therapies, well-designed prospective cohort studies with extended follow-up are urgently required to rigorously establish the safety profile of these natural products in this specific patient population.

## 5 Conclusion

The global incidence rate of IBD is rising, promoting the treatment strategy from immunosuppression to microbiota-centered intestinal homeostasis recovery. The gut microbiota and its SCFA metabolites play a crucial role. SCFAs transmit signals through GPR41/GPR43, inhibit HDACs, and activate AMPK, thereby regulating barrier function, immune response, oxidative stress, and tumor surveillance. IBD patients generally exhibit a decrease in SCFA-producing bacteria, including *F. prausnitzii*, *Roseburia*, and *Eubacteria rectale*, while fecal SCFAs levels are also low. These changes are associated with increased barrier permeability, immune dysfunction, and chronic inflammation.

Natural products such as polyphenols, polysaccharides, alkaloids, and herbal formulas may help modulate this axis. They exert a prebiotic-like effect, promoting the proliferation of SCFA-producing bacteria, endogenous SCFA synthesis, and restoring SCFA-GPR signaling. However, most of the available evidence remains preclinical, and the clinical relevance of these microbiota–SCFA-related effects remains to be established.

Taken together, natural products represent promising candidates for further investigation as modulators of the gut microbiota–SCFA axis in IBD. Their therapeutic application is constrained by interindividual variability, dose-response relationships, bioavailability, and limited clinical and long-term safety evidence. Further well-designed clinical studies are needed to determine their efficacy, optimal dosing, safety, and potential patient populations most likely to benefit.
